# Coffee consumption and prostate cancer risk: further evidence for inverse relationship

**DOI:** 10.1186/1475-2891-11-42

**Published:** 2012-06-13

**Authors:** Kashif Shafique, Philip McLoone, Khaver Qureshi, Hing Leung, Carole Hart, David S Morrison

**Affiliations:** 1 Institute of Health & Wellbeing, College of Medical, Veterinary and Life Sciences, Public Health, University of Glasgow, 1 Lilybank Gardens, Glasgow, G12 8RZ, UK; 2 West of Scotland Cancer Surveillance Unit, University of Glasgow, 1 Lilybank Gardens, Glasgow, G12 8RZ, UK; 3 Urology Department, Gartnavel General Hospital, 1053 Great Western Road, Glasgow, G12 0YN, UK; 4 Beatson Institute for Cancer Research, Garscube Estate, Switchback Road, Bearsden, Glasgow, G61 1BD, UK

**Keywords:** Coffee, Prostate cancer, Incidence, Gleason grade, Risk factor

## Abstract

**Background:**

Higher consumption of coffee intake has recently been linked with reduced risk of aggressive prostate cancer (PC) incidence, although meta-analysis of other studies that examine the association between coffee consumption and overall PC risk remains inconclusive. Only one recent study investigated the association between coffee intake and grade-specific incidence of PC, further evidence is required to understand the aetiology of aggressive PCs. Therefore, we conducted a prospective study to examine the relationship between coffee intake and overall as well as grade-specific PC risk.

**Methods:**

We conducted a prospective cohort study of 6017 men who were enrolled in the Collaborative cohort study in the UK between 1970 and 1973 and followed up to 31st December 2007. Cox Proportional Hazards Models were used to evaluate the association between coffee consumption and overall, as well as Gleason grade-specific, PC incidence.

**Results:**

Higher coffee consumption was inversely associated with risk of high grade but not with overall risk of PC. Men consuming 3 or more cups of coffee per day experienced 55% lower risk of high Gleason grade disease compared with non-coffee drinkers in analysis adjusted for age and social class (HR 0.45, 95% CI 0.23-0.90, p value for trend 0.01). This association changed a little after additional adjustment for Body Mass Index, smoking, cholesterol level, systolic blood pressure, tea intake and alcohol consumption.

**Conclusion:**

Coffee consumption reduces the risk of aggressive PC but not the overall risk.

## Introduction

Coffee is one of the most popular beverages worldwide and has been investigated for its role in PC development. Coffee compounds, diverse biologically active ingredients including caffeine, minerals and phytochemicals, have very strong metabolic, physiological, cellular and molecular effects [1]. Higher consumption of coffee for longer duration has been associated with improved glucose metabolism and insulin levels [2]. Also, growing evidence suggests that higher coffee consumption reduce the risk of diabetes mellitus [3,4], affect insulin like growth factors [5] and may alter the sex hormones [6,7]. Higher levels of circulating sex hormones, insulin like growth factors and insulin resistance are strongly associated with increased risk of PC [8-11]. Given, the beneficial effects of coffee consumption in lowering insulin resistance, insulin like growth factors and altering sex hormones may have some role in development of PC.

Epidemiological evidence on coffee consumption and PC risk remains equivocal. A meta-analysis of twelve epidemiological studies, including eight case–control and four prospective cohorts on the role of coffee in PC risk remained inconclusive [12]. There was a significant positive (harmful) association between higher coffee consumption and PC risk in case–control studies (RR 1.21, 95% CI 1.03-1.43), whereas, no significant relationship was observed in cohort studies (RR 0.97, 95% CI 0.68-1.38) [12]. A more recent meta-analysis of five cohort studies suggested a reduced risk of PC (RR 0.79, 95% CI 0.61-0.98) among coffee drinker compared with non-drinkers [13]. Most of the epidemiological studies had smaller number of cases [14,15], retrospective study design [16-19] and all lacked information on disease stage and grade.

Interestingly, a recent study on coffee consumption and PC risk from a large prospective cohort [20] reported a weak inverse association with overall risk and a strong inverse association between higher coffee intake and aggressive as well as lethal PC [20]. Authors concluded that higher coffee consumption can not be recommended to prevent PC and further epidemiological evidence on the association is required before making any recommendations. We therefore, investigated the association between coffee consumption and risk of overall as well as grade-specific PC from a large prospective cohort study with long follow up.

## Materials and Methods

The Collaborative study was the second of the Midspan studies which began in the 1960s and 1970s in Scotland, UK [21]. In brief, the Collaborative study was conducted on employed men and women aged from 21 to 75 years from 27 workplaces in Glasgow, Clydebank and Grangemouth between 1970 and 1973 [22]. The response rate was 70% for these workplaces for which response rates were available (87% of the sample).

Study protocols consisted of a self-administered questionnaire followed by a screening examination at a specially set-up clinic. Questions included demographic details, occupation and lifestyle habits. Daily coffee intake reported by the participants was categorised into three groups based on approximately equal number of participants in each group and also into two categories of coffee drinkers and non coffee drinkers. Weekly reported alcohol consumption was categorised by spirits, beer, and wine. This was converted to units of alcohol by taking one measure of spirits as 1 unit, 1 pint (0.6 litres) of beer as 2 units, and one bottle of wine as 6 units [23]. Four categories of alcohol consumption were formed (none, 1–10, 11–21, > 21 units of alcohol a week). As parts of the screening examination, measurements were made for height, weight and blood pressure. A blood sample was obtained at baseline screening for the measurement of total circulating plasma cholesterol. Body Mass Index (BMI) was calculated from weight (in kg) divided by height (in metres) squared and categorised according to the World Health Organisation classification in which BMI < 18.50 is underweight, 18.50 to < 25 is the normal range, 25 to <30 is overweight and ≥30 is obese. We combined the underweight and normal BMI categories because the number of subjects was small in the underweight category and only one subject developed PC. Social class was derived from occupation according to the relevant version of the General Register Office Classification of Occupation [24] and graded into six categories:

I (professional), II (intermediate), III non-manual (skilled non-manual), III manual (skilled manual), IV (partly skilled) and V (unskilled) [24]. Ex-smokers were defined as reporting giving up smoking at least a year before screening, otherwise they were defined as current smokers. Only the records for male participants were used for this study.

Follow up for mortality was carried out by flagging Midspan participants with the National Health Service Central Register. Information on cancer registrations and hospital activity was obtained by linkage to the Scottish Morbidity Records (SMR) data [25]. Follow up began on the date of screening to the date of cancer incidence, date of death, date of embarkation (leaving the United Kingdom) or the censor date of 31st December 2007, whichever came first.

### Ethical approval

The Privacy Advisory Committee of the Information Services Division of NHS Scotland gave permission for the linked data to be used in this study.

### Outcome definitions

PC was defined as International Classification of Diseases (ICD) revision 9 codes 185 and ICD-10 codes C61. PC incidence was ascertained if it appeared in any of the records from cancer registration (SMR06), acute hospital record (SMR01) or death record. Where a patient had PC recorded on more than one type of record, the earliest date was taken as time of first diagnosis. The Gleason grading system is a method used to distinguish the morphology of clinical PC to provide prognostic information. Gleason score was recorded from 1st January 1997 and therefore the analysis of grade-specific associations between coffee and PC was restricted to the follow up of the surviving cohort as of that date.

### Statistical analysis

For coffee consumption categories, Cox proportional hazards models were used to estimate hazard ratios (HRs) for PC incidence from screening, and for specific histological grade from 1st January 1997. Separate models were run for each Gleason category and men with PC and other Gleason scores were censored at their date of diagnosis. All analyses were conducted using STATA (stcox in STATA v11 StataCorp, College Station, TX, USA). Initial models were adjusted for age and social class. We then included smoking status, BMI, alcohol intake, coffee consumption, cholesterol level and systolic blood pressure in multivariate models. There were missing data for some covariates: 11 in social class; 41 in cholesterol level; 1 in height and weight; 1 in smoking status; and 1 in tea consumption. Total missing data for all covariates was less than 0.01% which did not change any of the associations when we ran the analysis both including those observations after imputations and excluding these individuals. We presented the final results after imputations in which missing information on continuous variables was replaced by the sample mean while for categorical variables missing data were replaced by modal values. The lowest category was used as referent for the coffee intake and all other categorical covariates. Adherence to the proportional hazards assumption was investigated by plotting smoothed Schoenfeld residuals against time; no violations of the assumption were identified. All statistical tests were two tailed and statistical significance was taken as p < 0.05.

## Results

In total, data for 6022 men were available from the Collaborative Cohort Study for this analysis; five study participants were lost to follow up so they were excluded from the analysis. Our final sample comprised 6,017 men followed-up for a total of 155,383 person-years. The median follow-up period was 28 years, maximum 37 years. Median age was 48 years at the time of screening (range, 21–75 years). The mean follow-up time between screening and PC diagnosis was 25.8 (SD 10.58) years.

Three hundred and eighteen men with PC were identified. Among 186 cancers that occurred from 1997 onward, 70 (37.6%) were high grade (Gleason score ≥ 8), 38 (20.4%) were intermediate grade (Gleason = 7), 41 (22.0%) low grade (Gleason ≤ 6) and the remaining 37 (19.9%) were of unknown Gleason score. Individuals who had a normal BMI, higher social class and smokers were more likely to consume three or more cups of coffee per day while those taking high amount of alcohol were more likely to be low or non-coffee drinkers (Table 1).

**Table 1:** Baseline characteristics of 6017 male participants of the Collaborative cohort study at screening and prostate cancer outcome

	**Categories of cups of coffee/day**					
	**0**		**1--2**		**≥ 3**	
Sample Size	2,628		2,059		1,330	
Person-years of follow up	65,051		54,603		35,728	
*Number of Deaths%(n)*	70.3	(1848)	64.0	(1317)	62.0	(825)
*Number of men with prostate cancers%(n)*	5.3	(139)	5.5	(114)	4.9	(65)
*Gleason grade ≥8*	48.2	(39)	29.9	(20)	28.9	(11)
*Gleason grade =7*	14.7	(12)	20.9	(14)	31.6	(12)
*Gleason grade <7*	21.0	(17)	25.4	(17)	18.4	(7)
*Unknown*	16.1	(13)	23.8	(16)	21.1	(8)
Mean Age (s.d.)	48.2	(7.3)	47.5	(7.2)	46.8	(7.3)
Mean Height (s.d.)	171.7	(6.9)	173.7	(7.1)	174.4	(6.8)
Mean Weight (s.d.)	74.3	(10.7)	75.9	(10.7)	76.2	(10.2)
Mean BMI (s.d.)	25.2	(3.2)	25.1	(3.0)	25.0	(2.9)
*BMI (kg m*^*-2*^*),%(n)*						
<25 (Under & Desirable)	48.7	(1281)	49.3	(1015)	50.5	(671)
25 – <30 (Overweight)	44.3	(1163)	45.3	(932)	44.8	(596)
≥30 (Obese)	7.0	(184)	5.4	(112)	4.7	(63)
*Alcohol intake (units/week),%(n)*						
0	24.2	(873)	29.7	(623)	36.4	(396)
1—10	30.7	(606)	31.6	(683)	26.4	(451)
11—21	22.0	(545)	19.8	(409)	20.2	(259)
> 21	23.2	(604)	18.9	(344)	17.1	(224)
*Smoking,%(n)*						
Never smoker	18.4	(483)	19.4	(400)	15.9	(211)
Smoker	60.5	(1590)	53.0	(1091)	61.1	(812)
Ex-smoker	21.1	(555)	27.6	(568)	23.1	(307)
*Social Class,%(n)*						
I&II	18.3	(480)	41.0	(847)	51.1	(680)
IIIN	29.0	(763)	15.1	(310)	11.0	(147)
IIIM	17.9	(470)	20.4	(417)	17.1	(227)
IV&V	34.8	(915)	23.5	(485)	20.8	(276)

We found no convincing association between coffee and overall hazard of PC (Table 2), nor any consistent relationship within low and intermediate grade disease (Table 3). We also conducted further analyses excluding individuals diagnosed with PC in the first 10 years of screening (n = 14), after multivariate adjustments the inverse association between coffee consumption of ≥3 cups/day and reduced PC risk slightly changed (HR = 0.80, 95% CI 0.59-1.10, p value 0.18) although remained non-significant.

**Table 2:** Multivariate hazard ratios (HR) for overall prostate cancer by coffee consumption in 6017 men

		**All Prostate Cancers**								
	**Total**	**n**	**Hazard Ratio (95% CI)**^**a**^			**p value**	**Hazard Ratio (95% CI)**^**b**^			**p value**
*Cups of coffee/day*										
0	2,628	(139)	1				1			
1--2	2,059	(114)	0.89	(0.69,	1.16)	0.39	0.95	(0.72,	1.24)	0.70
≥3	1,330	(65)	0.81	(0.60,	1.09)	0.18	0.93	(0.66,	1.31)	0.66
Cups of coffee (continuous)	6,017	(318)	0.95	(0.87,	1.02)	0.12	0.96	(0.81,	1.13)	0.64
*Age at screening*										
< 40	823	(30)	1				1			
40-49	2,621	(129)	1.85	(1.24,	2.76)	0.003	1.73	(1.16,	2.59)	0.008
50-59	2,424	(150)	4.49	(3.01,	6.68)	<0.001	4.13	(2.74,	6.22)	<0.001
60-75	149	(9)	9.28	(4.36,	19.73)	<0.001	8.21	(3.80,	17.72)	<0.001
*Cholesterol level*	6,017	(318)	1.02	(0.91,	1.14)	0.71	1.01	(0.91,	1.13)	0.80
*Systolic blood pressure*	6,017	(318)	1.00	(0.99,	1.01)	0.83	0.99	(0.99,	1.01)	0.57
*BMI (kg m*^*-2*^*)*										
<25 (Under & Desirable)	2,967	(152)	1				1			
25 – <30 (Overweight)	2,691	(148)	1.10	(0.88,	1.38)	0.41	1.11	(0.88,	1.40)	0.39
≥30 (Obese)	359	(18)	1.25	(0.77,	2.05)	0.37	1.28	(0.77,	2.11)	0.34
*Alcohol intake (units/week)*										
0	1,892	(115)		1				1		
1--10	1,740	(102)	0.97	(0.74,	1.27)	0.83	0.99	(0.75,	1.30)	0.92
11--21	1,213	(52)	0.83	(0.60,	1.15)	0.28	0.87	(0.62,	1.22)	0.42
> 21	1,172	(49)	0.94	(0.67,	1.32)	0.71	0.98	(0.69,	1.39)	0.92
*Smoking*										
Never smoker	1,094	(68)	1				1			
Smoker	3,492	(136)	0.92	(0.71,	1.27)	0.59	0.93	(0.69,	1.26)	0.65
Ex-smoker	1,430	(114)	1.43	(1.05,	1.93)	0.02	1.43	(1.05,	1.94)	0.02
*Social Class*										
I&II	2,007	(122)	1				1			
IIIN	1,220	(68)	0.88	(0.64,	1.22)	0.45	0.97	(0.68,	1.39)	0.88
IIIM	1,114	(75)	1.14	(0.85,	1.54)	0.38	1.16	(0.85,	1.58)	0.34
IV&V	1,676	(53)	0.97	(0.73,	1.29)	0.83	1.02	(0.74,	1.39)	0.92

Prostate cancer cases =318.

a = adjusted for age and social class, b = adjusted for age at screening, cholesterol, systolic blood pressure, BMI, alcohol intake, tea consumption, smoking status, social class. n = number of prostate cancer cases.

**Table 3:** **Hazard ratios for overall and Gleason-specific prostate cancer by coffee consumption categories among 3527 men from the Collaborative cohort study who survived until 1**^**st**^**January 1997**

			**Cups of coffee per day**		***P-value for trend***
		**0**	**1--2**	**≥3**	
**All prostate cancer**					
	Total PC cases	81	67	38	
	Hazard Ratio (95% CI)^a^	reference	0.86 (0.62-1.20)	0.75 (0.50-1.11)	0.15
	Hazard Ratio (95% CI)^b^	reference	0.84 (0.60-1.21)	0.74 (0.47-1.16)	0.23
**Gleason < 7**					
	Total PC cases	17	17	07	
	Hazard Ratio (95% CI)^a^	reference	1.18 (0.59-2.35)	0.75 (0.30-1.87)	0.65
	Hazard Ratio (95% CI)^b^	reference	1.04 (0.51-2.17)	0.54 (0.19-1.57)	0.48
**Gleason = 7**					
	Total PC cases	12	14	12	
	Hazard Ratio (95% CI)^a^	reference	1.13 (0.51-2.50)	1.39 (0.60-3.22)	0.45
	Hazard Ratio (95% CI)^b^	reference	1.23 (0.53-2.84)	1.79 (0.69-4.62)	0.17
**Gleason 8-10**					
	Total PC cases	39	20	11	
	Hazard Ratio (95% CI)^a^	reference	0.52 (0.30-0.91)	0.45 (0.23-0.90)	0.01
	Hazard Ratio (95% CI)^b^	reference	0.51 (0.28-0.92)	0.47 (0.22-1.01)	0.03
**Unknown Gleason**					
	Total PC cases	13	16	08	
	Hazard Ratio (95% CI)^a^	reference	1.26 (0.59-2.69)	0.99 (0.40-2.49)	0.08
	Hazard Ratio (95% CI)^b^	reference	1.17 (0.52-2.64)	0.88 (0.31-2.48)	0.89

Prostate cancer cases =186.

a = adjusted for age and social class, b = adjusted for age at screening, cholesterol, systolic blood pressure, BMI, alcohol intake, tea intake, smoking status, social class.

Interestingly, the hazard reduced significantly from the no coffee consumption to the higher category of coffee intake (HR 0.45, 95% CI 0.23-0.90) among aggressive PC (Gleason score ≥ 8) after adjustment for age and social class. These associations changed a little after additional adjustment for BMI, smoking, tea consumption, alcohol intake, plasma cholesterol and systolic blood pressure and a progressive reduction in risk of high grade PC remained between no coffee consumption and other two categories of coffee intake (p value for trend 0.03). However, the results were borderline significant which could be due to smaller number of cases (n = 11) in the highest category of coffee drinkers. We further investigated this association by combining the coffee categories as coffee drinkers and non-drinkers. Men who consumed any amount of coffee had significantly reduced risk of aggressive PC (HR 0.51, 95% CI 0.29-0.87, p = 0.01) compared with non-coffee drinkers after adjustments for age, BMI, smoking, social class, tea consumption, alcohol intake, plasma cholesterol and systolic blood pressure (data not shown).

## Discussion

We found that increased coffee intake was associated with reduced risk of aggressive PC but no significant relationship with either low grade (Gleason < 7), intermediate grade (Gleason = 7) and overall risk of developing PC in this population-based prospective cohort study. The men who drank 3 or more cups of coffee had 55% lower risk of developing aggressive disease (Gleason > 7). The overall null association between coffee consumption and PC incidence was consistent with many epidemiological studies [17-19]. Furthermore, inverse association between coffee and aggressive PC was fairly consistent with recently reported findings from Health Professional Follow-up Study, where a risk reduction of 47% was observed among high coffee consumers [20]. We are unaware of any other studies reporting the grade-specific PC risk in relation to coffee consumption.

The demographic and lifestyle characteristics of high coffee drinkers i.e. higher social class, desirable BMIs and current smoking, make it unlikely that confounding is a major explanation for these findings. Confounding by smoking and other lifestyle factors would bias the results towards null rather than an inverse association, which we report. It might be hypothesised that reverse causality might explain the protective effect of coffee on PC reported elsewhere. That is, men with undiagnosed PC may reduce their fluid intake, including coffee, to reduce symptoms of polyuria. Thus, PC would be less frequent among men who drank more coffee. However, PC often produces no urinary symptoms because most of the tumours arise in the peripheral zone of the prostate gland [26]. In addition, we conducted analyses to assess possible reverse causation by excluding individuals who were diagnosed in the first 10 years of screening, interestingly the association between coffee and overall risk of PC, exaggerated after excluding men who were diagnosed in first 10 years of follow-up, although remained non-significant.

Several biological mechanisms have been proposed which may explain the inverse association between high coffee consumption and aggressive PC. Coffee consumption can lower the insulin-like growth factor-1 (IGF-1) [5] which is associated with increased incidence of PC particularly high grade and advance stage disease [10,27]. Additionally, coffee is considered as a major source of antioxidants and many observational studies suggested that coffee consumption lower the inflammation [28,29]. There is growing evidence which suggests that inflammation play a vital role in the development of PC through the generation of proliferative inflammatory lesions [30]. Therefore, higher consumption of coffee may have a protective role in aggressive PC by reducing the inflammatory activity in tumour.

Our findings are based on a prospective cohort study in the United Kingdom, with long follow up (median: 28 years, max. 37 years), lower losses to follow up (0.01%), adjustment for other lifestyle habits and information on Gleason grades of the diagnosed cases. Our study has some limitations. First, the Collaborative study questionnaire did not have information on family history of PC and other dietary intake history including lycopene, multivitamins, processed meat and calcium which have been linked with the risk of PC in many studies [31-33]. Second, we relied on self reported coffee consumption at the time of screening which may suffer from information bias. Third, although reverse causation did not appear to explain our results, we can not rule it out as a possible source of bias. Fourth, PSA testing has remarkably influenced the incidence of PC in most Western countries including the United Kingdom, so any differences in low grade and high grade PC incidence may be because of screening differences between coffee drinkers and non-drinkers. Unfortunately, current cancer registry does not hold the information on PSA and we could not assess the screening differences between groups, which may explain the observed association. Sixth, the number of cases was small in this study, however this study had a fairly large sample compared to earlier prospective studies on coffee consumption and PC. Although grade-specific analysis was based on a smaller sub-group of cases, only one earlier study has investigated the coffee intake in relation to grade-specific PC incidence [20]. Given that the coffee consumption was assessed at baseline and the study had a fairly long follow-up, coffee intake among participants of this cohort may have changed considerably over time during the follow-up. This may have led to measurement error however; measurement error in this study is more likely to be non-differential and may have attenuated our results rather than exaggerated the association. Finally, any misclassification in coffee intake due to differences in cup size or type of coffee might bias our results. However, such misclassification would be expected to bias our results toward the null rather than the preventive association which we observed.

## Conclusion

In conclusion, men who consumed coffee regularly experienced a reduced risk of aggressive PC. Our findings are important, given the lack of modifiable risk factors for PC and particularly for high grade disease. Further studies are required to examine this association with different types of coffee and with evaluation of stage and grade specific risks.

## Competing interest

All authors declare that they have no conflicts of interest.

## Authors’ contributions

KS, PMcL, CH and DSM designed the study; KS and PMcL carried out statistical analyses; KS drafted the initial manuscript and all authors contributed to the final draft.
